# Modifications of the 5’ region of the CASPON^TM^ tag’s mRNA further enhance soluble recombinant protein production in *Escherichia coli*

**DOI:** 10.1186/s12934-024-02350-z

**Published:** 2024-03-20

**Authors:** Christoph Köppl, Wolfgang Buchinger, Gerald Striedner, Monika Cserjan-Puschmann

**Affiliations:** 1 Austrian Centre of Industrial Biotechnology, Muthgasse 18, Vienna, 1190 Austria; 2 Department of Biotechnology, Institute of Bioprocess Science and Engineering, University of Natural Resources and Life Sciences, Muthgasse 18, Vienna, 1190 Austria; 3 Biopharma Austria, Development Operations, Boehringer Ingelheim Regional Center Vienna GmbH & Co KG, Dr.-Boehringer-Gasse 5-11, Vienna, A-1121 Austria

**Keywords:** CASPON^TM^ technology, Fusion tag, 5’UTR, Recombinant protein, Rare codons, Free folding energy

## Abstract

**Background:**

*Escherichia coli* is one of the most commonly used host organisms for the production of biopharmaceuticals, as it allows for cost-efficient and fast recombinant protein expression. However, challenging proteins are often produced with low titres or as inclusion bodies, and the manufacturing process needs to be developed individually for each protein. Recently, we developed the CASPON^TM^ technology, a generic fusion tag-based platform process for high-titer soluble expression including a standardized downstream processing and highly specific enzymatic cleavage of the fusion tag. To assess potential strategies for further improvement of the N-terminally fused CASPON^TM^ tag, we modified the 5’UTR and 5’ region of the tag-coding mRNA to optimize the ribosome-mRNA interactions.

**Results:**

In the present work, we found that by modifying the 5’UTR sequence of a pET30a*cer* plasmid-based system, expression of the fusion protein CASPON^TM^-tumour necrosis factor α was altered in laboratory-scale carbon-limited fed-batch cultivations, but no significant increase in expression titre was achieved. Translation efficiency was highest for a construct carrying an expression enhancer element and additionally possessing a very favourable interaction energy between ribosome and mRNA (∆G_total_). However, a construct with comparatively low transcriptional efficiency, which lacked the expression enhancer sequence and carried the most favourable ∆G_total_ tested, led to the highest recombinant protein formation alongside the reference pET30a construct. Furthermore, we found, that by introducing synonymous mutations within the nucleotide sequence of the *T7AC* element of the CASPON^TM^ tag, utilizing a combination of rare and non-rare codons, the free folding energy of the nucleotides at the 5’ end (-4 to + 37) of the transcript encoding the CASPON^TM^ tag increased by 6 kcal/mol. Surprisingly, this new *T7AC*_*rare*_ variant led to improved recombinant protein titres by 1.3-fold up to 5.3-fold, shown with three industry-relevant proteins in lab-scale carbon limited fed-batch fermentations under industrially relevant conditions.

**Conclusions:**

This study reveals some of the complex interdependencies between the ribosome and mRNA that govern recombinant protein expression. By modifying the 5’UTR to obtain an optimized interaction energy between the mRNA and the ribosome (ΔG_total_), transcript levels were changed, highlighting the different translation efficiencies of individual transcripts. It was shown that the highest recombinant titre was not obtained by the construct with the most efficient translation but by a construct with a generally high transcript amount coupled with a favourable ΔG_total_. Furthermore, an unexpectedly high potential to enhance expression by introducing silent mutations including multiple rare codons into the 5’end of the CAPON^TM^ tag’s mRNA was identified. Although the titres of the fusion proteins were dramatically increased, no formation of inclusion bodies or negative impact on cell growth was observed. We hypothesize that the drastic increase in titre is most likely caused by better ribosomal binding site accessibility. Our study, which demonstrates the influence of changes in ribosome-mRNA interactions on protein expression under industrially relevant production conditions, opens the door to the applicability of the new *T7AC*_*rare*_ tag in biopharmaceutical industry using the CASPON^TM^ platform process.

**Supplementary Information:**

The online version contains supplementary material available at 10.1186/s12934-024-02350-z.

## Background

*Escherichia coli* is one of the most commonly used host organisms for the production of biopharmaceuticals, as it allows for cost-efficient and fast recombinant protein expression. Today, about 30% of approved biopharmaceuticals are produced by *E. coli*, and despite the recent advance of other production systems such as mammalian cell culture, new product approvals, like Beovu® and Cablivi^R^ in 2019, are constantly being granted, allowing *E. coli* to compete with both mammalian and non-mammalian hosts [1, 2]. *E. coli* is not equally suited for the expression of all proteins, as it lacks the ability for several key posttranslational modifications such as glycosylation, cytosolic disulphide bond formation and proteolytic protein maturation [3]. Nonetheless, there are certain commercially available strains that enable the formation of cytosolic disulphide bonds like the Shuffle and Origami *E. coli* hosts [3–5]. However, the expression of complex recombinant proteins in *E. coli* remains a major challenge as it can lead to accumulation of the protein of interest (POI) in inclusion bodies or low expression titres [6–9].

To obtain an efficient recombinant protein production process in *E. coli*, several factors play a key role. These include the basic choice of the expression system design, like plasmid-based or genome-integrated expression cassettes, and, in this regard, the copy number of the plasmid and the gene of interest (GOI) [2, 6]. Also, a suitable promotor system for high level protein expression must be employed and the mRNA has to have an appropriate stability for recombinant protein expression [6]. Furthermore, the choice of fermentation process and parameters, the media composition and induction strategy play a major role for high-yield recombinant protein production [6].

Alongside all these factors, the optimization of translation initiation, which is the rate limiting and most tightly regulated step in protein biosynthesis, has received much attention in the last two decades [10]. The translation initiation region (TIR), comprising the Shine-Dalgarno (SD) sequence, expression enhancers upstream of the SD sequence and the initiator codon, plays a key role for efficient translation of proteins [11]. Reis and Salis [12] created a model that describes and accurately predicts the translation initiation rate of bacterial genes based on the calculation of the total free binding energy (∆G_total_) of the 30 S ribosome complex to the ribosomal binding site (RBS) on the mRNA [12]. Several key factors are considered in this model including tRNA interactions at the start codon, hybridisation of the SD sequence with the anti-SD sequence, ribosome stretching and compressing facilitated by the spacer region, mRNA secondary structure, ribosome drafting, initial binding of the 30 S ribosomal subunit to upstream standby sites, unfolding of mRNA structures overlapping with the ribosomal footprint at the translational start position, as well as interactions with other mRNA binding proteins such as CsrA or Hfq [12–17]. This model test system can be used for accurate predictions and automated design of the bacterial RBS [17].

Expression enhancers are known to stimulate translation considerably by acting co-operatively with the SD sequence [11, 18, 19]. Originally discovered in the leader sequence of gene 10 in the T7 bacteriophage genome, which codes for a phage coat protein, numerous naturally occurring expression enhancers are known today [19]. Most commonly used expression enhancers carry an A/U-rich sequence upstream of the SD sequence, which interacts with the ribosomal S1 protein, and are capable of enhancing translation initiation by weakening the SD anti-SD sequence interaction, thus allowing for faster dissociation of the ribosome from the translation initiation site [18]. In vitro studies have shown to enhance the translation efficiency up to 16-fold upon incorporation of an expression enhancer at a suitable distance of 10 to 20 nucleotides upstream of the SD sequence [18].

Another way to enhance translation initiation is the modification of codons in the 5´end of the mRNA coding sequence (CDS) of a POI. Previous works investigating the influence of the nucleotide sequence encoding the POI have found the importance of codon usage at the 5´end of their CDS [20–24]. The exact region, where codon choice influences recombinant protein expression levels is still being debated, with different studies suggesting various regions, explicitly from nucleotide − 4 to + 37, the region centred around nucleotide + 10, the first 24 nucleotides and codon 2 to 8 respectively [20, 22–24]. However, the scientific literature is contradictory about what exactly causes variations in expression of endogenous *E. coli* proteins, their N-terminal regions fused to the green fluorescent protein (GFP) or GFP itself. Whereas Kudla et al. [20] and Goodman et al. [22] have stated that mRNA folding explains the highest share of variation in expression, Tuller et al. [21] found codon bias to be more strongly correlated to gene expression than folding energy. Tuller et al. [21] also investigated local translation efficiency and found a correlation between codon bias and protein-mRNA ratio, while free folding energy was not significantly correlated. Furthermore, it is important to point out that Goodman et al. [22] declared that even when combining influential factors such as promotor and RBS choice, secondary structure at the 5’end of the CDS, and GC content in a multiple linear regression, only 54% of the variation in expression level can be explained by the model. Similarly, Kudla et al. [20] stated that the free folding energy of nucleotides − 4 to + 37, despite being the most influential parameter tested, only explained 44% of the variation in reporter protein expression. This suggests, that there are additional and unknown effects governing gene expression [22]. The abovementioned experiments, focussing on the 5’ end of the CDS, have been performed in high throughput small scale cultivations at low cell densities only and the predictability of their results for industrially relevant fermentation conditions is still unknown, which makes further evaluation in C-limited fed-batch fermentation processes for biopharmaceutical industry necessary. Apart from that, the influence of codon bias, especially in the context of rare codons is not limited to the N-terminus of the CDS, as rare codons can aid in co-translational folding, as well as secretion and solubility of recombinant proteins, which has led to a paradigm shift from codon optimization to codon harmonization, aiming to replicate the codon usage pattern of the GOI in its original host rather than randomizing codon usage based on codon adaptation of the employed host organism [25–33].

We decided to explore the above-mentioned theses in the context of expression of POIs as fusion proteins, wherein the POIs are N-terminally tagged with a peptide tag. In previous studies, we have successfully developed the CASPON^TM^ platform technology, a generic combinatorial fusion tag-based platform process for the high-titre soluble expression of biopharmaceuticals in *E. coli* including a standardized downstream processing and highly specific enzymatic cleavage of the fusion tag, leaving the POI with its native N-terminus [34–37]. The CASPON^TM^ tag comprises an expression and solubility enhancing tag, *T7AC*, a His tag for IMAC purification and a cleavage site specific for a cp. caspase-2 variant. The CASPON^TM^ technology has been proven to be well suited for a variety of different biopharmaceutical proteins, outperforming previously published recombinant protein titres [35]. Despite the promising results obtained with the original CASPON^TM^ tag by testing numerous POIs, we observed POI-dependent differences in the expression levels, still leading to unsatisfying yields for some more challenging model proteins [35]. This prompted us to explore further means to improve the N-terminally fused CASPON^TM^ tag.

In this study, we examined several modifications of the 5’ region of the CASPON^TM^ tag’s mRNA by using two approaches: alteration of either (i) the 5’UTR or (ii) of the codon usage at the 5´end of the peptide tag. First, four different 5’UTR variants with varying interaction energies between RBS and ribosome (∆G_total_) were tested and the influence of an expression enhancer at an optimal distance to the SD sequence was evaluated. Then, we set out to optimize the 5’ end of the CDS of the N-terminal *T7AC* tag of the CASPON^TM^ tag for high titre expression by changing the codons of the first 12 amino acids of the published nucleic sequence of the *T7AC* tag, thereby creating the *T7AC*_*rare*_ tag. These modifications were tested on three different pharmaceutically relevant proteins. All constructs were evaluated using *E. coli* BL21(DE3) as production host, which was cultivated in laboratory-scale carbon limited fed-batch fermentations.

## Results

### Impact of 5’ UTR variations on CASPON^TM^-TNFα production and cell growth

**Fig. 1:**
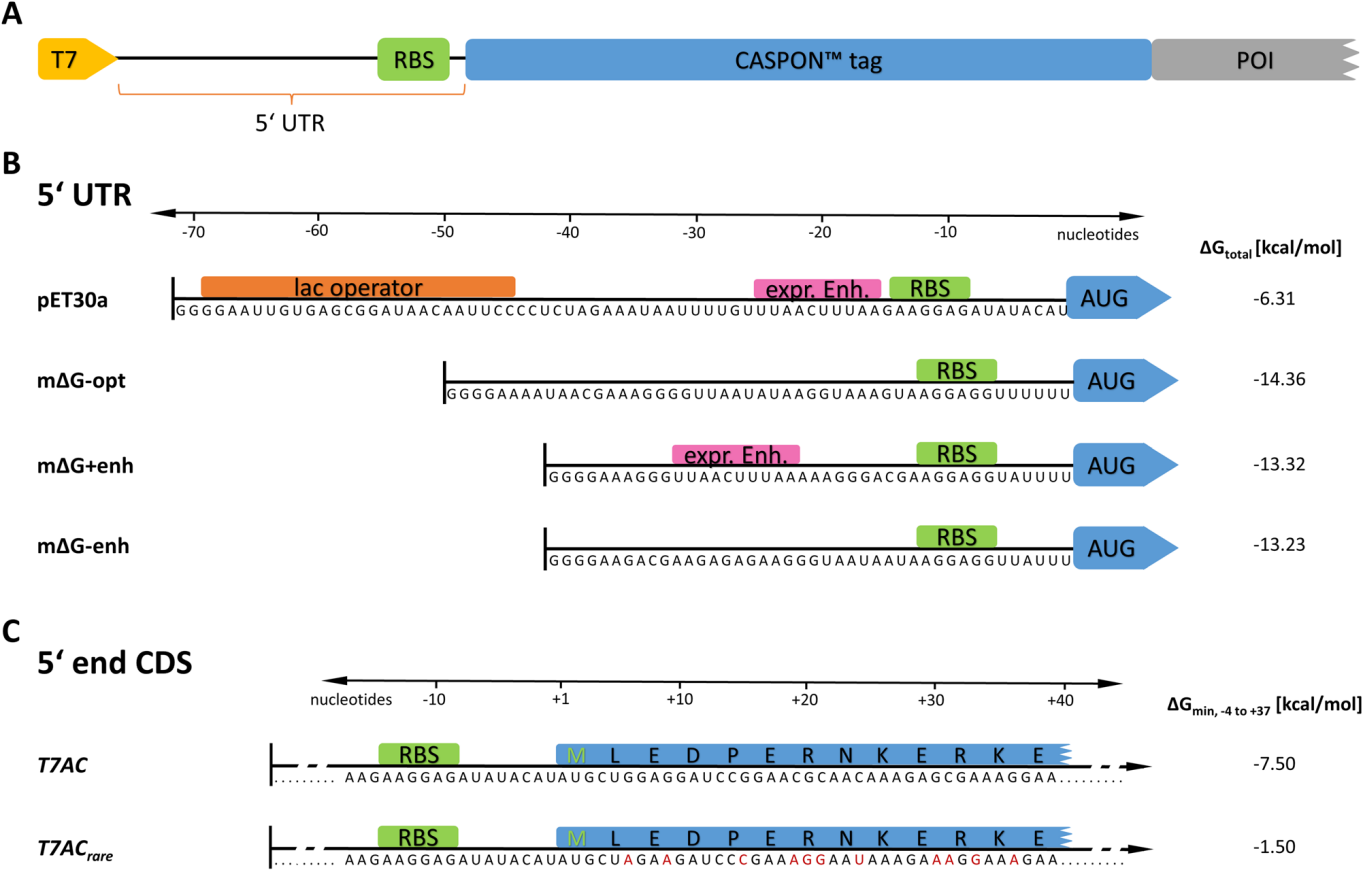
Schematic overview of tested constructs. (**A**): General structure of a bacterial 5’UTR in the context of the CASPON^TM^ platform construct. The T7 promotor is depicted as yellow arrow while the ribosomal binding site is illustrated as green rectangle. (**B**): Tested 5’UTR variants, their sequences and the corresponding total free interaction energies between ribosome and mRNA. Expression enhancer elements are depicted as pink rectangles. (**C**): N-terminal variants of the T7AC tag and their minimal free folding energies of nucleotides − 4 to + 37. The original *T7AC* tag sequence is depicted as well as the new *T7AC*_*rare*_ variant using a combination of rare and non-rare codons in its 5’end. Synonymous codons were chosen, which exhibit a low GC content as well as being classified as rare, with an occurrence lower than 10 per 1000 codons in the host cell genome, whereby the focus was on the latter parameter

To determine the impact of 5’UTR variations on recombinant protein production, we designed several 5’UTRs of varying interaction energies between mRNA and ribosome (∆G_total_) with and without expression enhancer elements (see Fig. 1B). The native sequence of the pET30a*cer* vector, which naturally carries a lac operator and an expression enhancer directly upstream of the RBS, served as reference with a free interaction energy (ΔG_total_) of -6.31 kcal/mol. The construct mΔG-opt was designed to maximize the interaction between mRNA and RBS using the algorithm developed by Reis et al. [12]. To achieve this, the length of the 5’UTR was set as flexible while lac operator and expression enhancer were omitted to maximise the degrees of freedom for the RBS design algorithm. This approach resulted in a construct with a very favourable ΔG_total_ of -14.36 kcal/mol. To test the combined influence of a low free energy of interaction (-13.32 kcal/mol) and the presence of an expression enhancer, construct mΔG + enh was designed to minimize ΔG_total_ while containing an expression enhancer sequence at a suitable distance of 10 bp to the RBS [18]. The sole effect of the expression enhancer was assessed using construct mΔG-enh as reference, which has a very similar interaction energy to construct mΔG + enh with − 13.23 kcal/mol but is lacking the expression enhancer element.

**Fig. 2:**
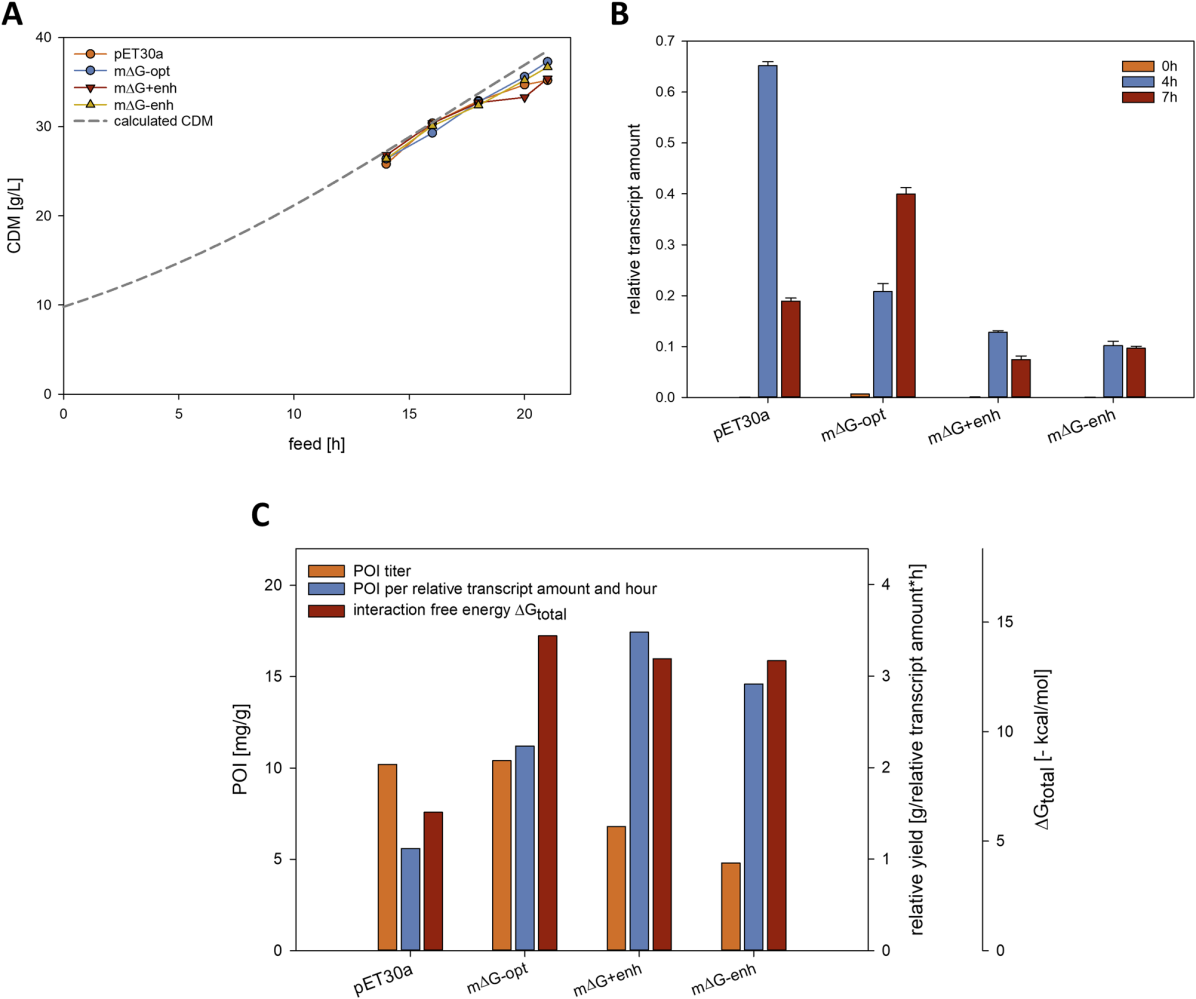
Results of the 5’UTR variant cultivations. (**A**): Bacterial cell growth kinetics of the different variants in the induction phase compared to the calculated theoretical growth curve. (**B**): Measured relative transcript amounts in relation to the 16 S rRNA at 0, 4 and 7 h after induction. (**C**): For each individual construct the recombinant protein titre at the end of fermentation, the product formed per relative transcript amount and hour, as well as the corresponding interaction free energy is depicted

The abovementioned constructs were cloned into the plasmid pET30a*cer* and transformed into the host strain BL21(DE3). In identical laboratory-scale carbon-limited fed-batch cultivations, we investigated all constructs regarding their influence on production strain growth (Fig. 2A), expression of CASPON^TM^-tumour necrosis factor α (TNFα) (Fig. 2C), as well as their transcript levels (Fig. 2B) immediately before and at two time points after induction.

The growth behaviour of the production hosts during the fermentations was comparable, as all variants almost reached the theoretical values of the calculated cell dry mass (CDM). The reference construct, named pET30a, housing the unchanged pET30a 5’UTR reached a biomass of 35.2 g CDM/L at the end of fermentation and produced 10.2 mg CASPON^TM^-TNFα/g CDM. Performance of the solely ∆G_total_ optimized construct (m∆G-opt) was very similar with a slightly higher end-of-fermentation biomass concentration of 37.3 g CDM/L and a recombinant protein titre of 10.4 mg/g CDM. Variant m∆G + enh, which carries an expression enhancer element additionally to possessing a favourable interaction energy between mRNA and ribosome, grew to a similar biomass of 35.4 g CDM/L and produced 6.8 mg/g CDM. The construct m∆G-enh carrying an almost identical ∆G_total_ to m∆G + enh but does not hold an expression enhancer element yielded 36.7 g CDM/L and produced 4.8 mg CASPON^TM^-TNFα/g CDM.

Relative transcript levels (CASPON^TM^-TNFα compared to host 16 S rRNA) of all four constructs were evaluated at 0, 4 and 7 h after induction (Fig. 2B). The transcript formation kinetics were highly different for the constructs pET30a and m∆G-opt compared to m∆G + enh and m∆G-enh. The unchanged pET30a 5’UTR led to a spike in transcript formation directly after induction, which declined to approximately a quarter of its original height after 7 h of induction. m∆G-opt 5’UTR showed a more consistent transcript formation kinetic with a constant rise in transcript levels after induction and a small amount of basal expression. The variants m∆G + enh and m∆G-enh showed the lowest and most stable levels of transcript formation in comparison. Although constructs m∆G + enh and m∆G-enh showed the lowest levels of relative transcript formation, we observed that the POI formation per relative transcript amount and hour was highest in m∆G + enh, followed by m∆G-enh, m∆G-opt and lastly the reference pET30a 5’UTR (Fig. 2C).

### Impact of codon variation at the CASPON^TM^ tag’s 5’ end on recombinant protein production and growth

**Fig. 3:**
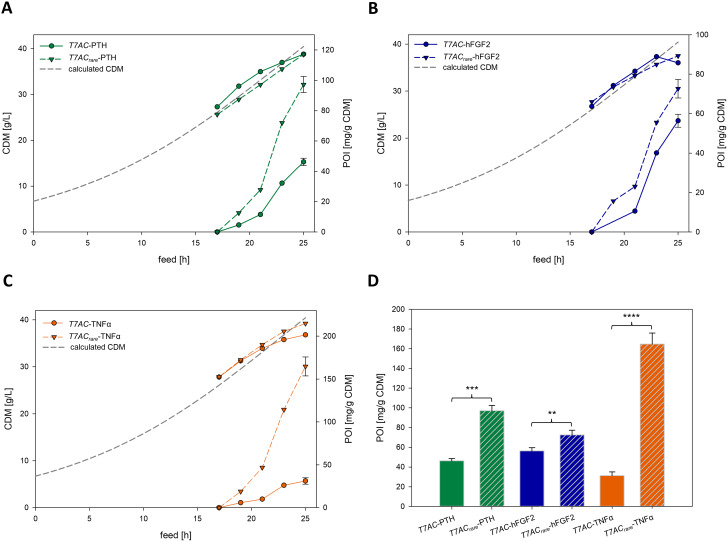
Growth behaviour and soluble product formation kinetics of *T7AC* and *T7AC*_*rare*_ tagged proteins in identical carbon-limited fed-batch fermentations. A to C show each a different protein. (**A**): PTH (**B**): hFGF2 (**C**): TNFα (**D**): direct comparison of specific recombinant protein titres at the end of fermentations. Solid bars represent the T7AC tagged variants, while hatched bars depict their *T7AC*_*rare*_ tagged counterparts. Results of statistical tests are notated with asterisks (* *p* ≤ 0.05, ** *p* ≤ 0.01, *** *p* ≤ 0.001, **** *p* ≤ 0.0001). Raw data as well as the results of the t-tests are summarized in the supplementary information Table S5. Data of all sampling points can be found in the supplementary information Table S4

To investigate the influence of codon variation at the 5’ end of the CASPON^TM^ tag’s mRNA on recombinant protein expression and cellular growth kinetics, we designed a construct, named *T7AC*_*rare*_, where we tested the codon usage with respect to the inclusion of codons exhibiting low GC content as well as being classified as rare, with an occurrence smaller than 10 per 1000 codons [38], whereas the focus was on the latter parameter. Compared to the original CASPON^TM^ tag encoding mRNA, this resulted in a rise of free folding energy of 6 kcal/mol at the mRNA nucleotide positions − 4 to + 37. This new *T7AC*_*rare*_ variant was then tested in a series of carbon limited fed-batch fermentations with three different pharmaceutically relevant proteins: parathyroid hormone (PTH), human fibroblast growth factor 2 (hFGF2) and TNFα (Fig. 3).

Cellular growth of both tag variants regardless of the expressed fusion protein was highly similar in all fermentations. Biomass formation followed the predicted values reasonably well (Fig. 3A to C) although the productivity for the fusion protein is significantly higher with *T7AC*_*rare*_. The theoretical CDM at fermentation end is 40.5 g/L. Measured CDM concentrations as well as specific and volumetric recombinant protein titres are summarized in Table 1.

**Table 1:** Measured CDM concentrations, soluble specific and volumetric recombinant protein titre at the end of fermentations as depicted in Fig. 3D. For additional data see supplementary information Table S4

	CDM [g/L]	Specific titre [mg/g CDM]	Volumetric titre [g/L]
*T7AC*-PTH	38.8	46.2	1.8
*T7AC*_*rare*_-PTH	38.7	97.2	3.6
*T7AC*-hFGF2	36.0	56.4	2.0
*T7AC*_*rare*_-hFGF2	37.5	72.7	2.7
*T7AC*-TNFα	36.8	31.2	1.1
*T7AC*_*rare*_-TNFα	39.2	164.8	6.5

Recombinant protein production was dramatically higher with the *T7AC*_*rare*_ tag compared to the unchanged *T7AC* tag for every tested protein. The comparative increase in recombinant fusion protein titre at the end of fermentation is dependent on the protein used. A 2.1-fold and 1.3-fold increase in specific titre was observed for PTH and hFGF2, respectively, while TNFα showed the biggest rise in titre with the *T7AC*_*rare*_ tag producing 5.3-fold as much TNFα as its reference.

## Discussion

The production of challenging recombinant proteins in *E. coli* remains a major challenge in industrial biotechnology [3]. Recently, we have reported the development of a universally applicable, generic fusion tag, the CASPON^TM^ tag, which does not only facilitate high soluble titres but also enhances recombinant protein production in *E. coli* [35]. Although the expression and solubility enhancing *T7AC* tag element within the CASPON^TM^ tag, which was derived from the T7 bacteriophage, led to satisfactory yields, we intended to further enhance the performance, especially for challenging proteins, by modifications of the nucleotide sequence in the 5’UTR and at the 5’ CDS end considering results of recently published studies [11, 20–22, 39]. Therefore, we examined the influence of free folding energy, the expression enhancing epsilon sequence [19] and mRNA-ribosome interactions in the 5’UTR. Regarding the 5’ end of the CDS of the *T7AC* element, the influence of a combination of rare and non-rare codons, selected for their rarity as well as low GC content, was tested.

Our 5’UTR variant experiment showed that although the pET30a reference had a comparatively unfavourable ∆G_total_, the resulting POI titer of the pET30a reference was nearly identical with the ∆G_total_ optimized variant m∆G-opt. This is very likely due to the nature of the T7 expression system coupled with the unchanged 5’UTR of the pET30a vector. As can be seen in Fig. 2B and C, the reference strain had the highest relative transcript amount directly after induction before dropping off, while also showing the highest total relative transcript amount. Since m∆G-opt produced less total relative transcript than the reference strain while still yielding roughly the same POI titer, it can be assumed that the unchanged pET30a vector with its expression enhancer sequence, in combination with the T7 expression system runs into limitations at a translational level. This becomes even clearer when we look at the translational efficiency per relative transcript level and time, as more transcript is needed in the reference strain to manufacture the same amount of protein as the m∆G-opt variant. While all variants except m∆G-opt showed constant or decreasing relative transcript levels at the end of fermentation, m∆G-opt was the sole construct with constantly increasing relative transcript levels throughout the induction phase. We hypothesize that this effect is caused by enhanced transcript stability compared to the other constructs. The lower transcript amounts produced by the altered 5’UTR variants compared to the pET30a reference could be explained by changes in the 5’UTR, which can lead to altered transcript levels and recombinant protein production [40, 41]. This effect was especially extreme in variants m∆G + enh and m∆G-enh where transcript production seems to be severely limited by the changes in the 5’UTR. However, the translational efficiency of both constructs outperformed the pET30a and m∆G-opt variants. Here the influence of the translation enhancer, which was incorporated into the m∆G + enh plasmid at an optimal distance to the start codon [18], can be clearly seen as the translational efficiency of the m∆G + enh construct is higher despite both constructs possessing highly similar ∆G_total_ values. Due to the nearly identical growth pattern in all constructs and the changes in transcript production, the pET30a and the m∆G-opt variants have proven to be the highest producing strains despite being the most inefficient regarding translation. To allow for a better comparison, a weaker expression system which produces constant amounts of transcript independently of the 5’UTR sequence would be needed.

Besides the free folding energy of the 5’CDS end as one determinant of translation efficiency, there are other factors such as codon bias, GC content, choice of ribosomal binding site as well as additional yet to be uncovered effects [20–22]. The unaltered *T7AC* nucleic acid sequence with its comparatively low free folding energy and the *T7AC*_*rare*_variant with higher free folding energy and comprising multiple rare codons (Fig. 1C) were evaluated with three different industrially relevant proteins. Contrary to previous studies, which reported that the introduction of rare codons into highly expressed genes reduce cellular fitness by depleting rare tRNA molecules and therefore change the translation efficiency of numerous genes, cell growth behaviour remained mostly unchanged [20, 42]. Upon enhancing the free folding energy of nucleotide positions − 4 to + 37 by 6 kcal/mol, substantial gains in recombinant protein yields were observed. It was unexpected that the use of rare codons within the newly designed 5´end of the *T7AC*_*rare*_ variant could so strongly enhance the expression of several POIs, as it has been previously stated that low translation rates caused by the use of rare codons lead to slow and inefficient product formation [43–45]. As previous studies have hypothesised, this effect is possibly caused by an increase in translation initiation efficiency, facilitated by an unstructured TIR, which may allow a more efficient ribosome entry [46]. Contradictory to previous literature where commonly a library of GFP variants was expressed in small scale cultivations and high throughput screening applied, our study focusses on the applicability in biopharmaceutical industry with three commercially relevant POIs [22, 24]. Our results describe a recombinant protein production increase of up to 5.3-fold when using our novel *T7AC*_*rare*_ sequence, where other translation efficiency investigating studies did not see such marked increase. For example, Goodman et al. [22] using GFP found a 4-fold median increase of fluorescence, while Voges et al. [24] reported an increase in GFP production of only greater than two-fold. Surprisingly, while drastic increases in recombinant protein production were observed when employing our novel *T7AC*_*rare*_ sequence, no inclusion bodies were formed. This suggests that the quality of recombinant protein did not change with the enhanced expression as is often the case [7]. Using our method, we substantially increased recombinant protein titres of three pharmaceutically relevant model proteins in lab-scale fed-batch fermentations, paving the way for its industrial application.

## Conclusions

In the present study, we showed that, among other key factors for recombinant protein expression, the optimization of the POI’s translation initiation, which is the rate limiting and most tightly regulated step in protein biosynthesis, is of great benefit. We examined the influence of several key factors for protein expression on cellular growth behaviour as well as recombinant protein production in carbon limited fed-batch fermentations, employing the previously designed CASPON^TM^ tag. Firstly, the influence of free folding energy, expression enhancing epsilon sequence and mRNA-ribosome interactions in the 5’UTR have been studied. Here we observed variations in transcript formation dependent on the 5’UTR sequence employed. Additionally, transcript efficiencies of the tested constructs varied substantially. The highest transcript efficiencies were observed in construct m∆G + enh carrying an expression enhancer at an optimal distance to the start codon as well as possessing a favourable ∆G_total_. However, the highest recombinant protein production was observed in construct m∆G-opt and the reference pET30a, despite both exhibiting a lower transcript efficiency. An unexpectedly high potential to enhance expression by introducing a combination of rare and non-rare codons into the 5’CDS end was identified. The use of these synonymous codons reduced the mRNA secondary structure and led to an increase in recombinant protein expression. Tested with three pharmaceutically relevant proteins, PTH, hFGF2 and TNFα, the recombinant protein production increased significantly ranging from 1.3-fold to 5.3-fold depending on the protein of interest in lab-scale fed-batch cultivations. As described by previous studies, the reduction of secondary structure present at the 5’ CDS end alone did not fully account for the observed variance in recombinant protein expression [20, 22]. Here we observed a similar outcome, specifically that the degree of expression enhancement using the *T7AC*_*rare*_ element varies for the tested POIs, despite having the same change in secondary structure.

Nonetheless, we assume that the expression enhancement is among other still unknown factors caused by better RBS accessibility for the ribosome. As modification of the codon usage in the POIs can lead to proteome-wide changes in translation efficiency in a codon-dependent manner, host cell fitness needs to be considered in production process development. Surprisingly, introduction of rare codons into the *T7AC*_*rare*_ element did not hamper cellular growth in our study, which is contrary to the findings of previous studies, where low codon adaptation especially in overexpressed genes is thought to be linked to decreased cellular fitness through the depletion of tRNA pools with low abundance, therefore hindering the translation of essential mRNAs [47]. Our results are especially unanticipated when viewed under the aspect of enhanced recombinant protein expression, as translation efficiency is thought to be directly linked to intracellular tRNA levels [43]. In an upcoming study, we will further investigate these effects, as well as test our hypothesis that more frequent translation initiation is more favourable than faster elongation by ribosome profiling approaches.

This study is a novelty in the field of translation efficiency investigating studies, as the POIs were produced in industrially-relevant fermentation conditions and are of pharmaceutical importance, while other studies have commonly used GFP as easy to monitor reporter protein [20, 22, 24]. Therefore, the new *T7AC*_*rare*_ tag is directly applicable for recombinant protein production in biopharmaceutical industry using the CASPON^TM^ platform process.

## Methods

### Strains

Strains used for recombinant protein expression as well as for cloning purposes were acquired from New England Biolabs (NEB, Ipswich, MA, USA). Chemically competent *E. coli* BL21(DE3) cells were used for all protein expression experiments, while chemically competent NEB-5α cells were applied for cloning purposes. Transformations and subsequent cultivations for cloning purposes were performed according to the manufacturer’s instructions.

### Design of 5’UTRs and calculation of minimal free folding energies

All tested 5’UTRs were designed *in silico* using the De Novo DNA software’s free energy RBS calculator [12–17, 48]. For that purpose, the original 5’UTR sequence of the pET30a*cer* vector was optimized according to the free energy model outlined below [48].

$$\eqalign{\Delta {G_{total}}\, = \,\Delta {G_{standby}}\, + \,\Delta {G_{mRNA - rRNA}} + \,\Delta {G_{spacing}}\\\, + \,\Delta {G_{start}} + \,\Delta {G_{stacking}}\, - \,\Delta {G_{mRNA}} \cr}$$

Since the sequence identity of the T7 promotor’s transcription start site has an influence on the initiation efficiency and specificity of the T7 RNA polymerase, nucleotides + 1 to + 6 of the 5’UTR were set to be identical with the original construct [49]. For creation of construct mΔG-opt, the algorithm was set to minimize ΔG_total_, therefore maximising mRNA – ribosome interactions. 5’UTR variant mΔG + enh was as well designed for a minimized ΔG_total_ with an additional expression enhancer sequence at a suitable distance from the SD sequence [18]. Lastly, mΔG-enh was created to closely match ΔG_total_ of construct mΔG + enh, lacking the expression enhancer sequence.

Minimal free folding energies (ΔG_min_) were calculated using the ViennaRNA Package (Version 2.6.3). The software calculates minimal free folding energies based on a loop-energy model combined with a dynamic prediction algorithm developed by Zuker et al. [50].This model calculates the total minimal free folding energy as the sum of all free folding energies contributed by the predicted loops in the mRNA sequence [50].

### Generation of expression constructs

Expression vectors were created using backbones from existing pET30a*cer* plasmids from a previous study and custom ordered inserts [35]. Q5® High-Fidelity DNA Polymerase, BsaI-HF®v2, DpnI and T4 DNA Ligase were purchased from NEB. Construction of the vector plasmids followed a standard cloning protocol. In brief, the plasmid backbone and insert, which was either ordered as oligonucleotide for small fragments or as gBlock for larger fragments, were amplified using polymerase chain reaction (pcr) using Q5® High-Fidelity DNA Polymerase. After amplification, both linear DNA fragments were purified using the Monarch® PCR & DNA Cleanup Kit (NEB) followed by restriction digest with BsaI-HF®v2 and DpnI (37 °C, overnight). After preparative agarose gel (2% agarose) purification (90 V, 90 min), the correctly sized bands were excised and dissolved using the Monarch® DNA Gel Extraction Kit (NEB). Ligation was performed at 16 °C overnight using T4 DNA Ligase (NEB) in a 1:3 molar ratio of backbone to insert and the ligation mix was directly transformed into chemically competent cells according to the manufacturer’s instructions. A comprehensive list of all primers and DNA fragments used can be found in the supplementary materials Table S1. All oligonucleotides labelled with the letter P were bought from Sigma Aldrich (Taufkirchen, Germany), while longer DNA sequences labelled with the letter G have been ordered as gBlocks from Integrated DNA Technologies (Coralville, IA, USA). After cloning, all constructs were sent for sequencing at Microsynth (Vienna, Austria) to confirm their correctness.

### Laboratory scale fed-batch fermentations

All fermentation procedures, as well as media preparation and composition were performed according to Fink et al. [51, 52]. Each experimental series (5’UTR variations and rare codon incorporations) had its own different fermentation setup (see supplementary materials Table S2 and S3). In each experimental series, the same fermentation process was repeated in an identical fashion for each construct. Briefly, cells were cultivated in the DASGIP parallel bioreactor system (Eppendorf SE, Hamburg, Germany) using a vessel with 2.1 L volume which allows for a maximum working volume of 1.8 L. To control relevant process parameters, the bioreactors were equipped with pH probes (Hamilton, Bonaduz, GR, Switzerland), optical dissolved O_2_ probes (Hamilton, Bonaduz, GR, Switzerland) and temperature probes. The dissolved oxygen concentration in the media was kept constantly at > 30% and the pH was sustained at 7.0 ± 0.2 by the addition of 12.5% ammonia solution. Temperature of the cultivation media was maintained at 37 ± 0.2 °C during the batch phase and shifted to 30 ± 0.2 °C at the beginning of the feed. Induction was facilitated by the introduction of 2 µmol Isopropyl-β-D-thiogalactopyranoside (IPTG) per gram of calculated CDM at the end of fermentation. All fermentation parameters not listed here can be found in the supplementary materials (Table S2 and S3). All computer-controlled fermentations processes were performed in single runs, the reproducibility of the fermentations has been shown in a previous study [35].

### Offline product analysis

Cell lysis and separation of the soluble und insoluble intracellular protein fractions was carried out according to Fink et al. [53]. Additionally, the cell lysis buffer contained 4 mmol L^− 1^ of NuPAGE Sample Reducing Agent (10x) (Invitrogen, Waltham, MA, USA). To estimate the recombinant protein concentration present in the cell lysate, reducing SDS-polyacrylamide gel electrophoresis (PAGE) was performed as described by Stargardt et al. and Cserjan-Puschmann et al. [37, 54]. Bovine Serum Albumin heat shock fraction (Sigma Aldrich, St. Louis, MO, USA) in the concentrations of 25, 50 and 75 µg/mL was used as quantification standard. SDS Page analysis of the samples was carried out in technical replicates according to the standards specified by Lingg et al. [55]. A brief description of the exact analytical procedure has been published by Köppl et al. [35]. Statistical testing of *T7AC* and *T7AC*_*rare*_ variants has been performed using the Welch’s t-test with a significance level α of 0.05. All raw data as well as the results of the t-tests are summarized in the supplementary information Table S5.

### RNA isolation, reverse transcription, and real time – quantitative PCR

Samples for RT-qPCR were processed according to Vazulka et al. [56]. Briefly, the samples were drawn of the carbon limited fed-batch cultivations at 0 h, 4 and 7 h after induction. Immediately, the cell suspension was mixed on ice with 0.5x its volume of 5% phenol in ethanol and centrifuged at 4 °C and 13 000 g for 2 min before being stored for further analysis at -80 °C. The sample volume was chosen to contain 3 mg CDM at the time of sampling according to the calculated theoretical CDM. RNA was isolated using the *Quick*-RNA Miniprep Kit (Zymo Research, Irvine, CA, USA) according to the manufacturer’s instructions. Immediately after RNA isolation, cDNA synthesis was carried out. For reverse transcription, SuperScript® IV Reverse Transcriptase (Thermo Fischer Scientific, Waltham, MA, USA) was used and the reaction was performed according to the protocol provided by the manufacturer. RT-qPCR reactions were carried out as described by Klanschnig et al. [57]. Briefly, for optimisation of the assay, aliquots of all reverse transcribed samples were pooled, and a dilution series prepared, which was used for generating standard curves for all employed primers. Results of primer testing can be found in the supplementary materials figure S1 to S4. iQ^TM^ SYBR® Green Supermix (Biorad, Hercules, CA, USA) was used according to the manufacturer’s instructions for all RT-qPCR reactions. A list of all employed RT-qPCR primers can be found in the supplementary materials Table S6. The MiniOpticon^TM^ (Biorad) system in combination with the CFX Manager^TM^ software (version 3.1.1517.0823) has been used to run and analyse all experiments. Calculations of the transcript ratios were carried out according to Carleton et al., specifically formula number 3 [58]. All quantifications were performed in technical triplicates.

### Electronic supplementary material

Below is the link to the electronic supplementary material.

Supplementary Material 1

## Data Availability

All data generated or analysed during this study are included in this published article and its additional files.
