# Supplementary material for: Modifications of the 5’ region of the CASPONTM tag’s mRNA further enhance soluble recombinant protein production in Escherichia coli
Source: Microb Cell Fact. 2024 Mar 20;23:86. doi: 10.1186/s12934-024-02350-z (PMC10953258; doi:10.1186/s12934-024-02350-z)
Supplement: Supplementary file 1 — Supplementary Material 1 [file 12934_2024_2350_MOESM1_ESM.docx]

**SUPPLEMENTARY INFORMATION**

Modifications of the 5’ region of the CASPON™ tag’s mRNA further enhance soluble recombinant protein production in *Escherichia coli*

Christoph Köppl ^1,2^, Wolfgang Buchinger ^3^, Gerald Striedner ^1,2^ and Monika Cserjan-Puschmann ^1,2, *^

^1^ Austrian Centre of Industrial Biotechnology, Muthgasse 18, 1190 Vienna, Austria;

^2^ Department of Biotechnology, Institute of Bioprocess Science and Engineering, University of Natural Resources and Life Sciences, Muthgasse 18, 1190 Vienna, Austria;

^3^ Biopharma Austria, Development Operations, Boehringer Ingelheim Regional Center Vienna GmbH & Co KG, Dr.-Boehringer-Gasse 5-11, A-1121 Vienna, Austria

# Oligonucleotide sequences

**Table S1**: Primer and gBlocks used for cloning and sequencing. Lower case letters denote BsaI cleavage sites with accompanying inserted bases for specific overhang creation.

| Primer/gBlock number | Name | Sequence (5’-3’) |
| --- | --- | --- |
| P1 | Seq-MCS-for | GAAGCAGCCCAGTAGTAG |
| P2 | Seq-MCS-rev | CGGATATAGTTCCTCCTTTCAG |
| P3 | Insert-contr | ATAGGGGAAGACGAAGAGAGAAGGGTAATAATAAGGAGGTTATTTATGCTGGAGGATCCGGAACGCAACAAAGAGCGAAAGGAAGCTGAGTTG |
| P4 | Insert-contr-for | ggtctcaATAGGGGAAGACGAAG |
| P5 | Insert-contr-rev | ggtctctctCAACTCAGCTTCCTTTC |
| P6 | Backbone-contr-for | ggtctcatgCAAGCTCAAACCGCTGA |
| P7 | Backbone-contr-rev | ggtctctctatAGTGAGTCGTATTAATTTCGC |
| P8 | Insert-Strep-TNFa-for | ggtctcaatGTCCGTTCCAGCAG |
| P9 | Insert-Strep-TNFa-rev | ggtctctcgGGCTTTGTTAGCAGCC |
| P10 | Backbone-Strep-TNFa-for | ggtctcaccCGAAAGGAAGCTGAGTTG |
| P11 | Backbone-Strep-TNFa-rev | ggtctctacATCCGCCACATCCAC |
| P12 | gBlock-for | ggtctcaGTTTTGCGCCATTCGAT |
| P13 | gBlock-rev | ggtctctaacaGCACTTGAGAATAAATCAG |
| P14 | Backbone-for | ggtctcaTGTTCAAAGGTCAGGGTTG |
| P15 | Backbone-rev | ggtctctaaacCTTTCGCGGTATGGCAT |
| P16 | T7AC-RARE-for | ggtctcaGAAAGGAATAAAGAAAGGAAA GAAGCTGAGTTGCAAG |
| P17 | T7AC-RARE-rev | ggtctcttttcGGGATCTTCTAGCATATGTATATCTCCTTC |
| G1 | gBlock-opti | ggtctcaGTTTTGCGCCATTCGATGGTGTCCGGGATCTCGACGCTCTCCCTTATGCGACTCCTGCATTAGGAAGCAGCCCAGTAGTAGGTTGAGGCCGTTGAGCACCGCCGCCGCAAGGAATGGTGCATGCAAGGAGATGGCGCCCAACAGTCCCCCGGCCACGGGGCCTGCCACCATACCCACGCCGAAACAAGCGCTCATGAGCCCGAAGTGGCGAGCCCGATCTTCCCCATCGGTGATGTCGGCGATATAGGCGCCAGCAACCGCACCTGTGGCGCCGGTGATGCCGGCCACGATGCGTCCGGCGTAGAGGATCGAGATCGATCTCGATCCCGCGAAATTAATACGACTCACTATAGGGGAAAATAACGAAAGGGGTTAATATAAGGTAAAGTAAGGAGGTTTTTTATGCTGGAGGATCCGGAACGCAACAAAGAGCGAAAGGAAGCTGAGTTGCAAGCTCAAACCGCTGAGCAACACCATCATCACCATCATAGCGCGTGGAGCCATCCGCAGTTTGAAAAAGGCAGCGGCGTGGATGTGGCGGATGTCCGTTCCAGCAGCCGTACGCCGAGCGATAAACCTGTCGCGCACGTAGTGGCGAATCCGCAAGCCGAGGGTCAGCTGCAGTGGCTGAATCGTCGCGCGAACGCGCTGCTGGCCAATGGTGTTGAGCTGCGTGACAACCAACTGGTTGTTCCATCCGAAGGCCTGTACCTGATTTATTCTCAAGTGCtgttagagacc |
| G2 | gBlock-ExpE | ggtctcaGTTTTGCGCCATTCGATGGTGTCCGGGATCTCGACGCTCTCCCTTATGCGACTCCTGCATTAGGAAGCAGCCCAGTAGTAGGTTGAGGCCGTTGAGCACCGCCGCCGCAAGGAATGGTGCATGCAAGGAGATGGCGCCCAACAGTCCCCCGGCCACGGGGCCTGCCACCATACCCACGCCGAAACAAGCGCTCATGAGCCCGAAGTGGCGAGCCCGATCTTCCCCATCGGTGATGTCGGCGATATAGGCGCCAGCAACCGCACCTGTGGCGCCGGTGATGCCGGCCACGATGCGTCCGGCGTAGAGGATCGAGATCGATCTCGATCCCGCGAAATTAATACGACTCACTATAGGGGAAAGGGTTAACTTTAAAAAGGGACGAAGGAGGTATTTTATGCTGGAGGATCCGGAACGCAACAAAGAGCGAAAGGAAGCTGAGTTGCAAGCTCAAACCGCTGAGCAACACCATCATCACCATCATAGCGCGTGGAGCCATCCGCAGTTTGAAAAAGGCAGCGGCGTGGATGTGGCGGATGTCCGTTCCAGCAGCCGTACGCCGAGCGATAAACCTGTCGCGCACGTAGTGGCGAATCCGCAAGCCGAGGGTCAGCTGCAGTGGCTGAATCGTCGCGCGAACGCGCTGCTGGCCAATGGTGTTGAGCTGCGTGACAACCAACTGGTTGTTCCATCCGAAGGCCTGTACCTGATTTATTCTCAAGTGCtgttagagacc |

# Fermentation parameters

***Table S2****: Fermentation conditions for all 5'UTR variations*

|  | **Temp. [°C]** | **CDM [g/L]** | **Vol. [mL]** | **Duration [h]** | **µ [h^-1^]** | **Generations** |
| --- | --- | --- | --- | --- | --- | --- |
| Batch | 37 | 9.8 | 500 | 8.5 | - | - |
| Fed- Batch | 30 | 38.6 | 1038 | 21.0 | 0.1 | 3.0 |

Induction was carried out at feed hour 14 with an IPTG pulse added directly into the bioreactor corresponding to 2 µmol IPTG per gram of biomass at the end of fermentation.

|  | **Temp. [°C]** | **CDM [g/L]** | **Vol. [mL]** | **Duration [h]** | **µ [h^-1^]** | **Generations** |
| --- | --- | --- | --- | --- | --- | --- |
| Batch | 37 | 6.7 | 500 | 7.5 | - | - |
| Fed- Batch | 30 | 40.4 | 1014 | 25.0 | 0.1 | 3.6 |

***Table S3:*** *Fermentation conditions for all rare codon experiments*

Induction was carried out at feed hour 17 with an IPTG pulse added directly into the bioreactor corresponding to 2 µmol IPTG per gram of biomass at the end of fermentation.

Table S4: Summarizing data of Figure 3

| T7AC-PTH | CDM [g/L] | feed [h] | spec. titer [mg/g CDM] | SD [mg/g CDM] | vol. titer [g/L] | SD [g/L] |
| --- | --- | --- | --- | --- | --- | --- |
|  | 27.3 | 17 | 0.0 |  | 0.0 |  |
|  | 31.8 | 19 | 4.6 |  | 0.1 |  |
|  | 35.0 | 21 | 11.5 |  | 0.4 |  |
|  | 37.0 | 23 | 32.2 |  | 1.2 |  |
|  | 38.8 | 25 | 46.2 | 2.4 | 1.8 | 0.09 |
|  |  |  |  |  |  |  |
| T7ACrare-PTH | **CDM [g/L]** | **feed [h]** | **spec. titer [mg/g CDM]** | **SD [mg/g CDM]** | **vol. titer [g/L]** | **SD [g/L]** |
|  | 25.6 | 17 | 0.0 |  | 0.0 |  |
|  | 28.9 | 19 | 12.5 |  | 0.4 |  |
|  | 32.1 | 21 | 27.8 |  | 0.9 |  |
|  | 35.5 | 23 | 71.9 |  | 2.5 |  |
|  | 38.7 | 25 | 97.2 | 5.3 | 3.6 | 0.20 |
|  |  |  |  |  |  |  |
| T7AC-hFGF2 | **CDM [g/L]** | **feed [h]** | **spec. titer [mg/g CDM]** | **SD [mg/g CDM]** | **vol. titer [g/L]** | **SD [g/L]** |
|  | 26.7 | 17 | 0.0 |  | 0.0 |  |
|  | 31.2 | 19 |  |  |  |  |
|  | 34.2 | 21 | 10.6 |  | 0.4 |  |
|  | 37.3 | 23 | 40.1 |  | 1.5 |  |
|  | 36.0 | 25 | 56.4 | 3.3 | 2.0 | 0.12 |
|  |  |  |  |  |  |  |
| T7ACrare-hFGF2 | **CDM [g/L]** | **feed [h]** | **spec. titer [mg/g CDM]** | **SD [mg/g CDM]** | **vol. titer [g/L]** | **SD [g/L]** |
|  | 27.7 | 17 | 0.0 |  | 0.0 |  |
|  | 30.9 | 19 | 15.7 |  | 0.5 |  |
|  | 33.3 | 21 | 23.1 |  | 0.8 |  |
|  | 35.7 | 23 | 55.6 |  | 2.0 |  |
|  | 37.5 | 25 | 72.7 | 4.7 | 2.7 | 0.18 |
|  |  |  |  |  |  |  |
| T7AC-TNFα | **CDM [g/L]** | **feed [h]** | **spec. titer [mg/g CDM]** | **SD [mg/g CDM]** | **vol. titer [g/L]** | **SD [g/L]** |
|  | 27.8 | 17 | 0.0 |  | 0.0 |  |
|  | 31.3 | 19 | 5.8 |  | 0.2 |  |
|  | 33.9 | 21 | 9.9 |  | 0.3 |  |
|  | 35.8 | 23 | 26.1 |  | 0.9 |  |
|  | 36.8 | 25 | 31.2 | 3.8 | 1.1 | 0.14 |
|  |  |  |  |  |  |  |
| T7ACrare-TNFα | **CDM [g/L]** | **feed [h]** | **spec. titer [mg/g CDM]** | **SD [mg/g CDM]** | **vol. titer [g/L]** | **SD [g/L]** |
|  | 27.9 | 17 | 0.0 |  | 0.0 |  |
|  | 31.5 | 19 | 18.9 |  | 0.6 |  |
|  | 34.7 | 21 | 46.8 |  | 1.6 |  |
|  | 37.6 | 23 | 114.3 |  | 4.3 |  |
|  | 39.2 | 25 | 164.8 | 11.1 | 6.5 | 0.44 |

Table S5: Raw data and results of T7AC and T7AC_rare_ variant statistical testing

# RT-qPCR primer sequences and testing

| **Target** | **Direction** | **Sequence 5’-3’** | **Length [nt]** | **Primer efficiency** |
| --- | --- | --- | --- | --- |
| 16S rRNA | Reverse | CTGATTCACCGTGGCATTCT | 20 | 90.6% |
| 16S rRNA | Forward | CCTCATAAAGTGCGTCGTAGT | 21 | 90.6% |
| TNFα | Reverse | AATCCGGGCGGTTGATTT | 18 | 95.6% |
| TNFα | Forward | TGTTGCTGACCCATACCATTAG | 22 | 95.6% |

**Table S6**: Sequences and primer efficiencies of all primer pairs used for RT-qPCR


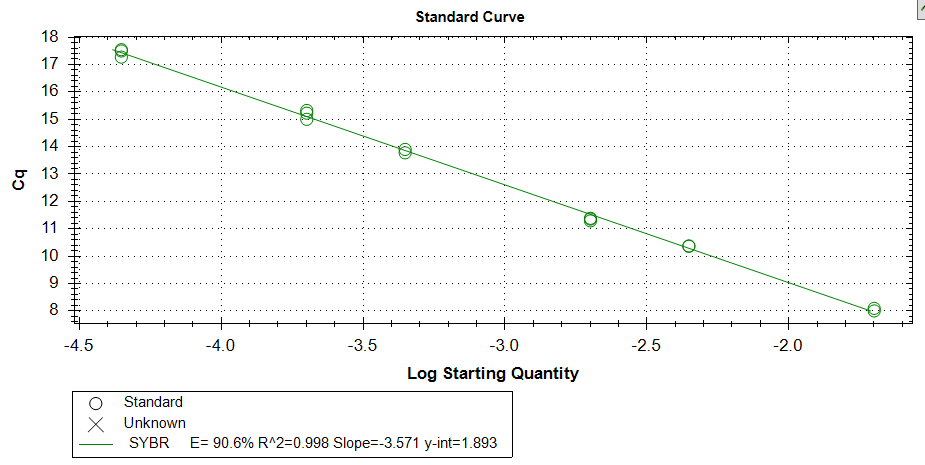


**
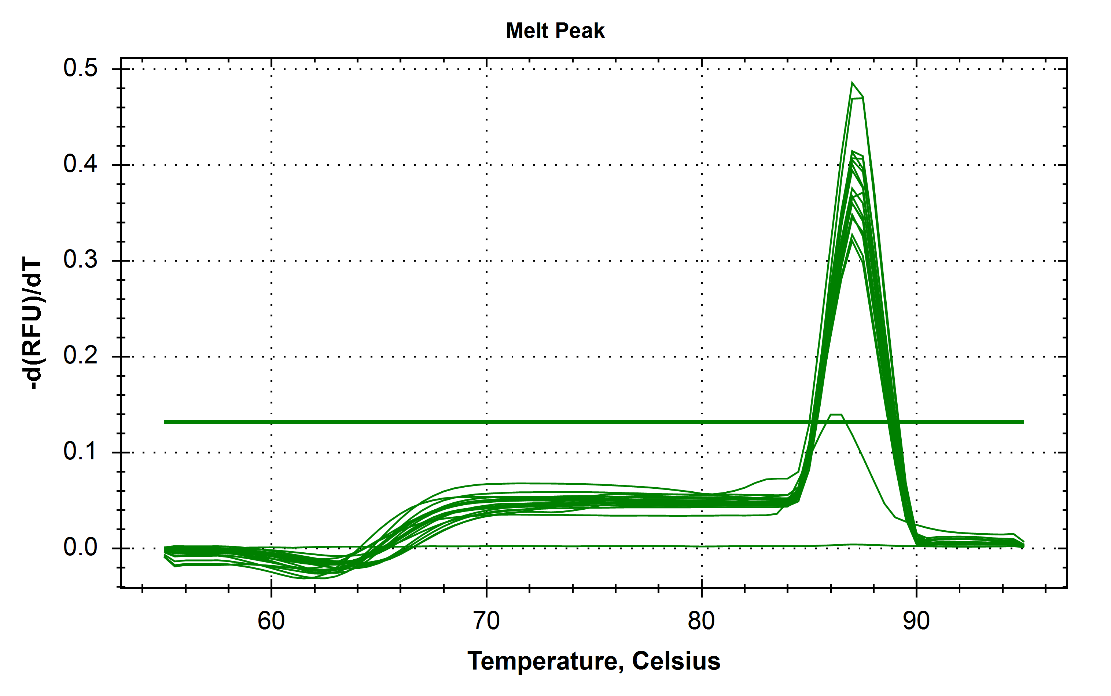
Figure S1:** Standard curve of 16S rRNA primer testing

**Figure S2**: Melting temperature analysis of 16S rRNA RT-qPCR products


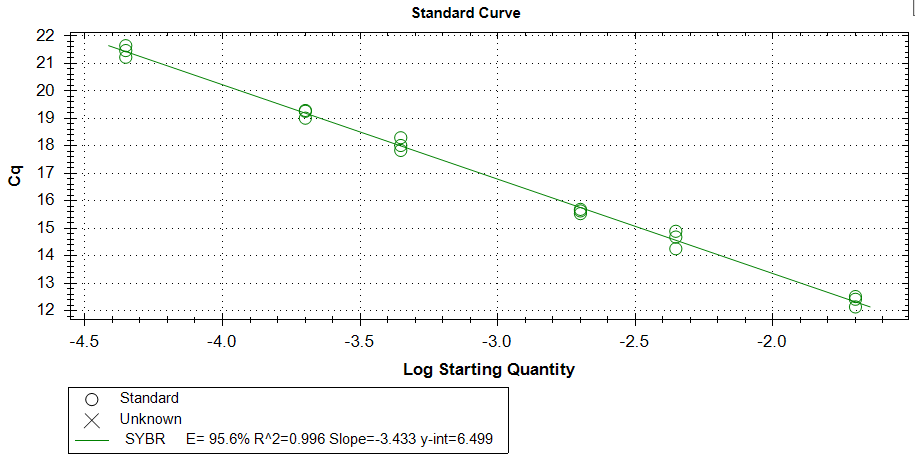


**Figure S3:** Standard curve of TNFα primer testing


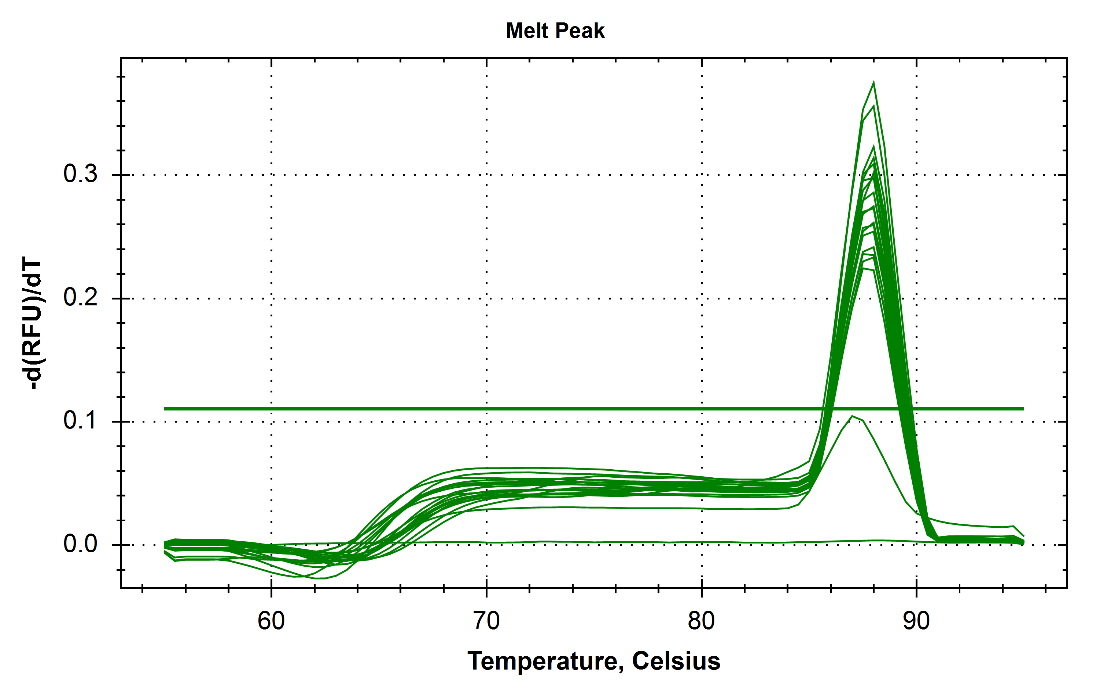


**Figure S4:** Melting temperature analysis of TNFα RT-qPCR products
